# Divergent susceptibilities to AAV-SaCas9-gRNA vector-mediated genome-editing in a single-cell-derived cell population

**DOI:** 10.1186/s13104-017-3028-4

**Published:** 2017-12-08

**Authors:** Salma G. Morsy, Jason M. Tonne, Yaxi Zhu, Brian Lu, Karol Budzik, James W. Krempski, Sherine A. Ali, Mohamed A. El-Feky, Yasuhiro Ikeda

**Affiliations:** 1 Department of Molecular Medicine, Mayo Clinic, College of Medicine, 200 First St. SW, Rochester, MN 55905 USA; 2 Center for Regenerative Medicine, Mayo Clinic, College of Medicine, Rochester, MN 55905 USA; 3 Cancer Biology, South Egypt Cancer Institute, Assiut University, Assiut, Egypt; 4 Microbiology and Immunology, Faculty of Medicine, Assiut University, Assiut, Egypt

**Keywords:** Adeno Associated viral vector, SaCas9 optimization, Off-target, Whole exome sequencing, Genome editing resistance

## Abstract

**Objective:**

Recombinant adeno-associated virus (AAV)-based vectors are characterized by their robust and safe transgene delivery. The CRISPR/Cas9 and guide RNA (gRNA) system present a promising genome-editing platform, and a recent development of a shorter Cas9 enzyme from *Staphylococcus aureus* (SaCas9) allows generation of high titer single AAV vectors which carry both saCas9- and gRNA-expression cassettes. Here, we used two AAV-SaCas9 vectors with distinct GFP-targeted gRNA sequences and determined the impact of AAV-SaCas9-gRNA vector treatment in a single cell clone carrying a GFP-expression cassette.

**Results:**

Our results showed comparable GFP knockout efficiencies (40–50%) upon a single low-dose infection. Three consecutive transductions of 25-fold higher doses of vectors showed 80% GFP knockout efficiency. To analyze the “AAV-SaCas9-resistant cell population”, we sorted the residual GFP-positive cells and assessed their permissiveness to super-infection with two AAV-Cas9-GFP vectors. We found the sorted cells were significantly more resistant to the GFP knockout mediated by the same AAV vector, but not by the other GFP-targeted AAV vector. Our data therefore demonstrate highly efficient genome-editing by the AAV-SaCas9-gRNA vector system. Differential susceptibilities of single cell-derived cells to the AAV-SaCas9-gRNA-mediated genome editing may represent a formidable barrier to achieve 100% genome editing efficiency by this vector system.

## Introduction

Targeted genome editing with a programmable nuclease enables diverse genome manipulation in a sequence-specific manner [1]. Recently, the CRISPR/Cas9 system has emerged as a robust RNA-guided genome editing tool [2] where type II CRISPR/Cas9 nucleases are adapted from the microbial adaptive immune defense system [1, 3]. Two main components are required for eukaryotic genome editing, a Cas9 enzyme and a chimeric short-guide RNA (gRNA) derived from CRISPR RNA and a non-coding Trans activating crRNA (tracr RNA) [4, 5]. CRISPR/Cas9 recognizes its target through the gRNA with a 20 nucleotide sequence and a protospacer adjacent motif (PAM) which directs Cas9 to a specific DNA target site through RNA–DNA complementarity base-pairing [6, 7]. Cas9 endonucleases can then produce a site-specific double-strand break on the target sequence. The CRISPR/Cas9 enzyme from *Streptococcus pyogenes*, SpCas9, has been widely used for robust genome-editing applications. Although the CRISPR/SpCas9 system is highly versatile and efficient in targeted genome editing and has many advantages over other gene editing tools such as TALEN and ZNF [8, 9]; however, its clinical application has been limited due to undesirable off target effects [10].

Viral vectors are a promising approach for delivery of Cas9 in vitro and in vivo [11]. Among them, the adeno-associated viral (AAV) vector system provides a versatile, non-integrating gene delivery platform characterized by its non-pathogenic, low immunogenic transduction of dividing and non-dividing cells [12]. However, its packaging capacity has limited the use of AAV vectors to transfer the commonly used SpCas9-gRNA system. Recently, a 1 kb shorter form of Cas9 from *Staphylococcus aureus*, SaCas9, was identified to show comparable genome editing efficiency to SpCas9. Use of SaCas9 allows generation of a high titer, single AAV vector, which carries both SaCas9- and gRNA-expression cassettes [4, 13, 14]. In contrast to the widely used SpCas9 system, the characteristics of the AAV-SaCas9-gRNA vector-mediated genome editing system have not been extensively characterized. In this study, we characterized the biological properties of the AAV-SaCas9-gRNA vector system using GFP-targeting vectors and GFP-expressing cell clones.

## Main text

### Materials and methods

#### Cells

HT1080 (ATCC CCL-121) and 293T (ATCC CRL-11268) cells were maintained in Dulbecco’s Modified Eagle’s medium (HyClone, GE Healthcare, Pittsburgh, PA) supplemented with 10% fetal calf serum, 50 U/ml penicillin, and 50 μg/ml streptomycin [15].

#### Plasmids

pX601-AAV-CMV::NLS-SaCas9-NLS-3xHA-bGHpA;U6::BsaI-sgRNA was kindly provided by the Feng Zhang Lab (Addgene plasmid #61591). PX601 carrying human codon-optimized SaCas9 under control of CMV promotor and gRNA is driven by U6 promotor. We constructed two gRNA plasmids to target two different positions in the GFP open reading frame sequence, with GFP-targeted 20 nucleotides with the SaCas9 PAM sequence (NNGRRT). The AAV-Cas9-GFP#1 vector plasmid, pAAV-Cas9-GFP1, was constructed using the two complementary oligonucleotides (forward 5′-CACCGGGCAACATCCTGGGGCACAAGC-3′ and reverse 5′-AAACGCTTGTGCCCCAGGATGTTGCCC-3′, which targets the GFP sequence 5′-GGCAACATCCTGGGGCACAAGC-3′ in the sense direction. The second vector, AAV-Cas9-GFP#2 was, made with two complementary oligonucleotides; forward 5′-CACCGGCAAGGGCGAGGAGCTGTTCA-3′ and the reverse 5′-AAACGTGAACAGCTCCTCGCCCTTGCC-3′, which targets the GFP sequence 5′-GCAAGGGCGAGGAGCTGTTCAC-3′ in the sense direction. Each pair of oligonucleotides were phosphorylated and annealed according to Dr Zhang’s protocol and then cloned into the BsaI site in the pX601 plasmid [16], resulting in pAAV-Cas9-GFP1 and pAAV-Cas9-GFP2. The cloned gRNA sequences were verified by sequencing analysis using U6 forward primer 5′-CGAGGTAACCTTTCCCATGATTCCTT-3′.

#### AAV-SaCas9-gRNA vectors

Helper-free AAV2 vectors were produced in 293T cells, purified and titer as previously described [15, 17]. Specifically, we used AAV2 capsid expressing, pRep2Cap2, for the packaging plasmid, while pHelper was used as a helper plasmid (Stratagene).

#### Flow cytometry

Recombinant adeno-associated virus based vectors vector-treated HT1080 cells were harvested using trypsin (CORNING, Corning, NY), washed twice with PBS (HyClone), resuspended in 4% paraformaldehyde diluted in PBS. Fluorescence-activated cell sorting (FACS) were performed by using FACS Calibur (BD Biosciences, San Jose, CA). Fifty thousand events were captured for each sample. GFP-positive cell populations were analyzed by Flowjo_V10 (FlowJo, Ashland, OR).

#### Detection of GFP-targeted genome editing

Cells were harvested for total cellular DNA isolation by Genomic DNA Purification Kit (Thermo Fisher Scientific, Waltham, MA) according to the manufacturer’s instructions. Targeted sequence was PCR amplified using KOD Hot Start DNA polymerase (EMD Millipore, Billerica, MA). PCR amplified fragments were cloned into pCR™-Blunt II-TOPO^®^ Vector (Thermo Fisher Scientific) and then sequenced by the M13 forward primer. Data were analyzed by DNA Dynamo software for targeted genome editing.

#### Whole exome sequencing

Total DNA samples were isolated from HT1080-GFP#F cells transduced with either AAV2-Cas9-GFP#1 and GFP#2 or untreated controls, and subjected to the standard whole exome sequencing analysis. The enriched exome was sequenced by Illumina HiSeq2000 (Illumina, San Diego, CA) and analyzed by IGV program by the Mayo Genomics Core.

#### Statistical analysis

We employed unpaired Student’s *t* Test to assess the significance of data sets between two groups at the same vector dosage. Data were expressed as mean ± standard errors. Significance was accepted for the P-value less than 0.05.

### Results and discussion

#### Efficient genome editing by single AAV-saCas9-gRNA vectors

**Fig. 1:**
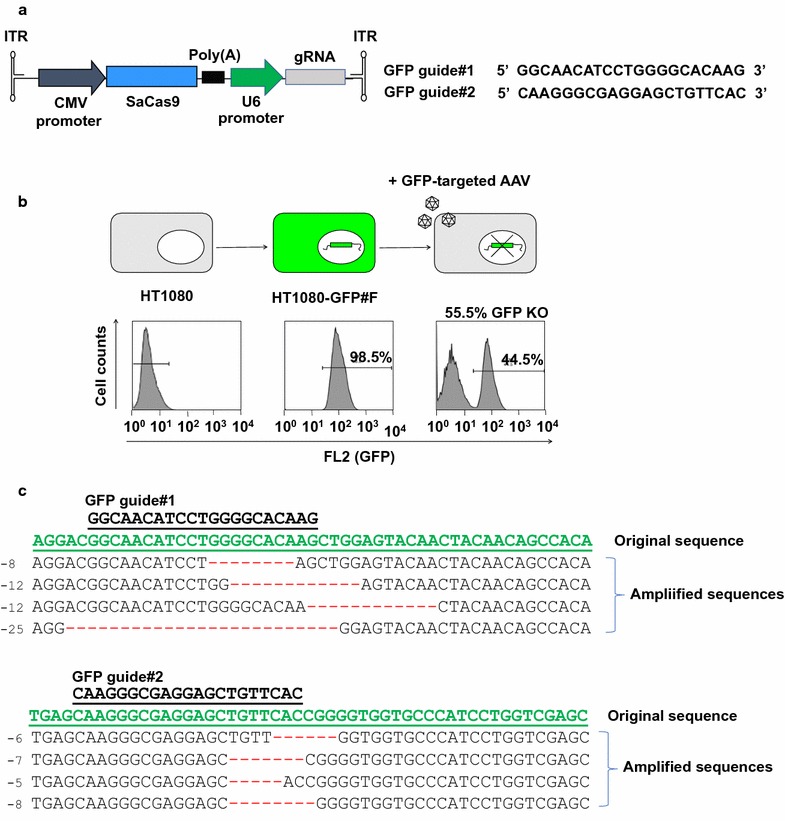
**a** Schematic representation of the AAV vector constructs used in this study. GFP-targeted SaCas9 AAV vectors were produced through introduction of 20 homologous nucleotide sequences for the target GFP sequences into the pX601 plasmid. SaCas9 and gRNA are expressed under the control of CMV and U6 promotors, respectively. **b** GFP expressing HT1080 single cell clones were generated by transduction with lenti-GFP-Puro vector at an MOI 0.0001, followed by puromycin selection and expansion of single cell clones. Fluorescence-activated cell sorting was performed to confirm GFP positive cell populations (middle panels). When GFP#F cells were transduced with AAV-Cas9-GFP1 and AAV-Cas9-GFP2 at MOI 2 × 10^4^, 42.7 and 55.5% GFP knockout efficiency was observed (right panels, n = 2). **c** Targeted genome editing was verified by sequencing. gRNA-targeted regions were amplified and sequenced. Deletions at the targeted sequences by gRNA#1 and #2 are shown with the original GFP sequences

We first established single cell-derived GFP-expressing cell clones by transducing HT1080 cells with a lentiviral vector expressing GFP and puromycin resistance at a multiplicity of infection (MOI) of 0.0001, followed by puromycin selection and expansion of selected single cell clones (Fig. 1a) [18, 19]. A stable GFP clone, designated GFP#F, was used in this study. FACS analysis confirmed over 98% of cells as GFP-positive (Fig. 1b for #F). To test the GFP-target genome editing efficiency of two AAV-CRISPR-SaCas9 vectors, GFP#F cells were transduced by the vectors at an MOI of 2 × 10^4^, which is a relatively high AAV vector dosage but does not cause notable toxicity for our in vitro study. 1 week after transduction, cells were analyzed by flow cytometry for GFP-positive cell populations. Transduction of AAV-Cas9-GFP1 and AAV-Cas9-GFP2 vectors resulted in 43 and 55% knockout efficiencies, respectively (Fig. 1b right panel for AAV-Cas9-GFP2, data not shown for AAV-Cas9-GFP1). To verify targeted genome editing by the AAV-mediated SaCas9 and gRNA delivery, total cellular DNA from vector-infected GFP#F cells were isolated and target GFP sequences were amplified by PCR, followed by cloning and sequencing of individual clones. As depicted in Fig. 1c, deletions in the gRNA-targeted regions were found in AAV-Cas9-GFP1- and AAV-Cas9-GFP2-treated cells.

#### Enhanced genome editing efficiency through multiple transductions

**Fig. 2:**
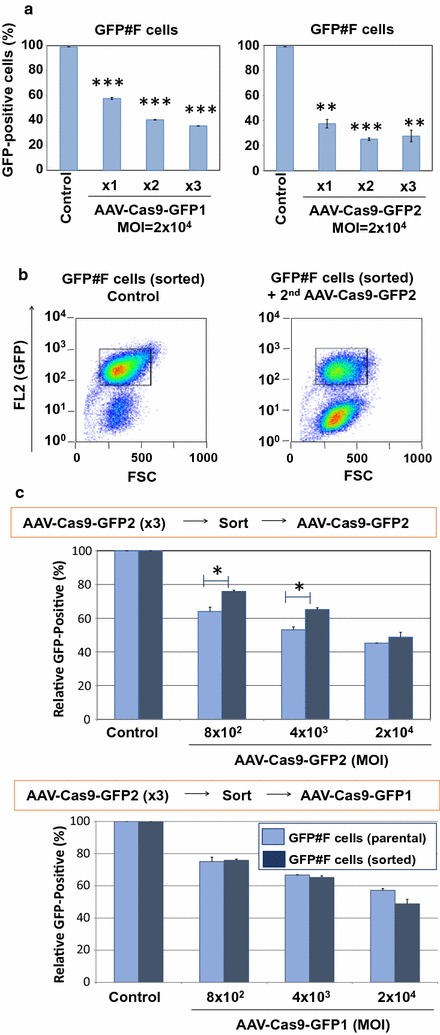
**a** GFP#F cells were transduced with two AAV vectors for three consecutive days at MOI 2 × 10^4^. When treated with AAV-Cas9-GFP1 (left panel), 57.7 ± 0.7, 40.6 ± 0.2 and 35.6 ± 0.1% cells were found GFP-positive, while 37.5 ± 3.3, 25.2 ± 1.1, 27.7 ± 4.5% of cells were found GFP-positive with AAV-Cas9-GFP2 (right panel) vector infection after one, two or three administrations, respectively. Error bars indicate STDEV. *P < 0.05, **P < 0.01, and ***P < 0.001. **b** We sorted the GFP-positive cell population in the GFP#F cells, which were treated by AAV-Cas9-GFP2 vector at MOI 2 × 10^4^ for three consecutive days. Sorted cells were used for AAV-Cas9-GFP vector superinfection study. Representative FACS images for sorted cells (left panel) and AAV-superinfected cells (right panel) were shown. **c** Parental GFP#F cells and sorted cells were transduced with AAV-Cas9-GFP2 at MOI of 8 × 10^2^, 4 × 10^3^, 2 × 10^4^ gc and GFP-positive cell populations were analyzed by FACS (upper panel). Lower panel shows the results of GFP-positive cell populations after super-infection with another GFP-targeting vector, AAV-Cas9-GFP1 vector. The averages of two independent experiments are shown. Error bars represent SEM (*P < 0.05)

The aforementioned studies demonstrated that GFP knockout efficiency can be improved by increasing AAV vector doses. We therefore transduced GFP#F cells with both AAV-Cas9-GFP1 and AAV-Cas9-GFP2 vectors at MOI 2 × 10^4^ gc for three consecutive days. Although we found increased genome editing efficiency by multiple transductions, a subset of cells appeared to be highly resistant to the saCas9-mediated gene editing as there was no significant difference between cells transduced with the vector twice or three times (Fig. 2a). To further assess the resistance of these cells to SaCas9-mediated genome editing, we sorted the residual GFP-positive cell population in the GFP#F cells which were transduced three times with AAV-Cas9-GFP2 at MOI 2 × 10^4^. Sorted GFP-positive #F cells and their parental GFP#F cells were then transduced with the same vector, i.e. AAV-Cas9-GFP2, at fivefold increasing doses (MOI 8 × 10^2^, 4 × 10^3^, 2 × 10^4^ gc). Flow cytometry analysis showed increasing GFP knockout patterns with increased vector doses in both parental and sorted cells. However, sorted cells had significantly higher numbers of GFP-positive cells than parental GFP#F controls (Fig. 2c, upper panel), indicating that sorted cells were more resistant to the same AAV vector-mediated genome editing. Intriguingly, both sorted and parental GFP#F cells were equally susceptible to the other GFP-targeted AAV vector, AAV-Cas9-GFP1 (Fig. 2c, lower panel). Those data suggest the existence of heterogeneous cell populations in single cell-derived cells which show differential susceptibilities to a particular gRNA-targeted genome editing, likely due to epigenetic modifications of the gRNA-targeted region [20, 21].

#### Off-targeted effects induced by AAV-SaCas9-gRNA vectors

**Fig. 3:**
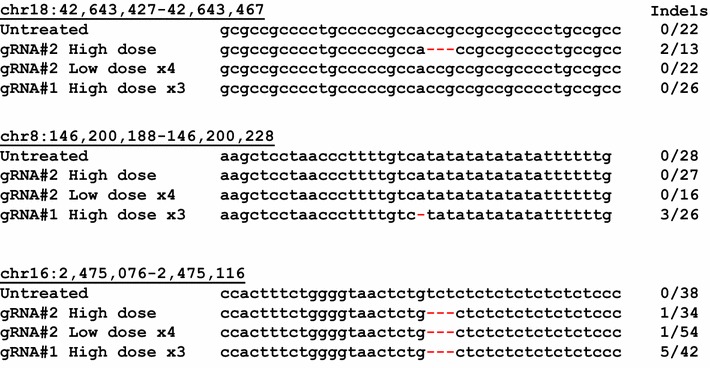
Three off-target deletions identified by exome-sequencing were shown. GFP#F cells were treated by AAV-Cas9-GFP2 vector at high dose (MOI = 2 × 10^4^ gc, × 1), AAV-Cas9-GFP2 vector at low dose four times (MOI = 8 × 10^2^ gc, × 4) or AAV-Cas9-GFP1 vector at high doses three times (MOI = 2 × 10^4^ gc, × 3) to get 70% GFP knockout. Total DNA samples from the three samples, along with DNA sample from uninfected control cells, were analyzed by exome-sequencing to identify off-targeted genome editing sites

To assess potential off-target effects of the AAV-SaCas9-gRNA vector system, we performed whole exome sequencing for genomic DNA which was extracted from GFP#F cells which showed approximately 70% GFP knockout after AAV vector infection under three different conditions; (i) a single, high dose transduction by AAV-Cas9-GFP2 (MOI = 2 × 10^4^ gc), (ii) multiple, low dose transductions by AAV-Cas9-GFP2 (MOI = 8 × 10^2^ gc, four times), and (iii) multiple, high dose transductions by AAV-Cas9-GFP1 (MOI = 2 × 10^4^ gc, three times). This allowed us to evaluate the differences in the off target effects according to different treatment conditions, including “single high dose vs. multiple low dose” by the same vector, as well as two different gRNAs vectors. Untreated GFP#F cells were included as control. Bioinformatic analyses comparing the reads with reference human exome sequence data identified a total of 670 loci as potential indels. We then performed multiple filtration steps. We first excluded the loci that had the same indels in both AAV-treated and untreated control samples. We also eliminated the candidate loci with changes in multiple nucleotide repeats (e.g. AAAAAAA to AAAAAA). We then eliminated the loci with less than 10 reads per sample. Using IGV program which allows assessment of individual sequencing data, we further verified the sequences. When the sequences of each locus among four samples were compared, we found three loci with deletions in AAV-vector-treated samples (Fig. 3). Those three loci showed no notable homologies to either gRNA sequences, suggesting induction of gRNA-independent off-targeted genome deletions by AAV-SaCas9-gRNA vectors. Although sample sizes are small, we found a trend that cells transduced with high dose vectors tended to show more off-targets than cells transduced with multiple low-dose treatments.

Here, we demonstrate efficient genome editing by the AAV-SaCas9-gRNA vector system, especially upon multiple high-dose transductions. Our data also suggest the presence of cell populations with divergent susceptibility to targeted genome-editing in single-cell-derived cells, which may represent a formidable barrier for complete genome editing by this vector system. Molecular mechanisms underlying this heterogeneity remain to be determined. Since consecutive day transduction enhanced genome editing efficiency, it is plausible that a subset of cells can become temporarily resistant to the vector-mediated genome editing, possibly depending on their cell cycle stages. Our cell sorting study also indicates the presence of a cell population which shows longer-term, gRNA-target-site-specific resistance. We postulate two mechanisms underlying this observation. One possibility is due to epigenetic modifications of the targeted site, which makes this locus more resistant to the vector-mediated genome editing than the same locus in other cells [20, 21]. Another possibility is the appearance of “escape mutant” cells with gRNA-target site modification. Our analysis of on-target genome modifications found various deletions in target regions, some of which were in-frame deletions of 6 and 12 nucleotides (Fig. 1c). These in-frame deletions will lead to deletions of 2 and 4 amino acid residues, which may not completely disrupt expression of GFP. We speculate that cells carrying such target-site-altered GFP sequence also played a role as “AAV-SaCas9-gRNA-resistant cells”. Delivery of multiple AAV vectors which target distinct regions in the same gene will likely reduce the appearance of “escape mutant cells” in target cell populations.

Studies have identified relatively high off-target effects of the CRISPR/Cas9 system [7, 22]. Earlier studies have focused on potential off-targeted editing on genome loci with high similarities to target sequences [7, 23]. However, this approach assesses a subset of potential off-target sites and could miss a much larger number of off-target sites in the entire genome [22]. Additionally, when compared to the SpCas9 system, potential off-target effects of the SaCas9-gRNA system have not been extensively studied. Here, we used an unbiased whole exome sequencing analysis and identified at least three off-target sites across the genome. We found a trend of increased off-target effects upon high dose AAV vector administration. Notably, those three loci showed no notable homology to the gRNA-target sequences, suggesting that the SaCas9-gRNA vectors can induce off-targeted genome editing in a gRNA-independent manner. We postulate that high levels of SaCas9 expression by AAV vectors increase gRNA-independent, non-specific SaCas9 binding to the genomic DNA. Recently, Zhang and colleagues have reported a high fidelity SaCas9 mutant with the R499A/Q500A/R654A/G655A mutations, which reduce the off-targeted effects of SaCas9 to undetectable levels [10]. The use of AAV vectors carrying the R499A/Q500A/R654A/G655A SaCas9 mutant may reduce the levels of off-targeted genome editing by this vector system.

### Limitations

We acknowledge the presence of some limitations in our study including that targeting GFP gene with CRISPR-Cas9 system results in deletions of 2 and 4 amino acid residues, which may not completely disrupt expression of GFP. Another notable limitation is that the study was conducted using only in vitro cells based on HT1080 cell line.

## Abbreviations

AAV: recombinant adeno-associated virus based vectors
CRISPR: Clustered Regularly Interspaced Short Palindromic Repeats
Cas: CRISPR-associated
CrRNA: CRISPR RNA
tracrRNA: trans-activating CRISPR RNA
sgRNA: single guide RNA
PAM: protospacer adjacent motif
SpCas9: *Streptococcus pyogenes* Cas9
SaCas9: *Staphylococcus aureus* Cas9
GFP: green fluorescent protein
MOI: multiplicity of infection
gc: genome copies
